# 

**Published:** 1921-07-16

**Authors:** 


July 16, 1921] [Price Twopence

### The Hospital

The Workers' Newspaper of Administrative Medicine and Institutional Life, Administration, National Insurance and Health.

### CONTENTS

**Doctors in the Witness-Box** ... ... ... ... 255

**The Sequel to the Mansion House Meeting** ... 256

**Notes of the Week** ... ... ... ... 257
Voluntary Hospitals Commission—Medical Men at Newcastle—The Various Types of Artificial Limbs—King George's Sanatorium for Sailors—The Late Sir G. H. Savage—Royal Westminster Ophthalmic Hospital—The Latest Hospital of Recovery—Mass Contributions—A Joint Policy at Bristol and Sheffield.

**The Progress of Psycho-Analysis** ... ... ... 259

**Reaction Against Fear...** ... ... ... ... 260

A Necessary Part of Preventive Medicine.

**The Financial Position of Guy's Hospital** ... 261
Present Considerations and Future Outlook.

**War Pensions Administration** ... ... ... 263

**The Question of Mass Contributions** ... ... 264

**The Public Well-Being ...** ... ... ... 265

Work for Disabled Soldiers—The Conference on Child Welfare.

**The Editor's Letter-Box** ... .. ... ... 267
Doctor and Patient: "The Letters of a Layman"; A Layman's Letter Answered; More Letters of a Layman: A Postscript; The Period Between School-Leaving and Hospital Training.

**Parliament and the Professions** ... ... ... 268

**The Matrons' and Sisters' Department** ... ... 269
The Sister-Tutor as an Aid to Economy.
The National Network of Nurses and Midwives.

**Round the Hospitals** ... ... ... ... 270

**The General Nursing Council for Scotland** ... 271

**The Association of Hospital Matrons** ... ... 272

**Hospital Sunday, 1921** ... ... ... ... ... 272

**Nurses' Registration Act, 1919** ... ... ... 272

**The College of Nursing (Limited by Guarantee)** 272

**Poor-Law Authorities on the Draft Syllabus** ... v

